# NMR Spectroscopic Identification of Urolithin G, a Novel Trihydroxy Urolithin Produced by Human Intestinal *Enterocloster* Species

**DOI:** 10.1021/acs.jafc.3c01675

**Published:** 2023-07-26

**Authors:** David Beltrán, María D. Frutos-Lisón, Rocío García-Villalba, José E. Yuste, Victor García, Juan C. Espín, María V. Selma, Francisco A. Tomás-Barberán

**Affiliations:** † Quality, Safety and Bioactivity of Plant-Derived Foods, CEBAS-CSIC, University Campus, Edif. 25, Espinardo, 30100 Murcia, Spain; ‡ Metabolomics Unit, CEBAS-CSIC, 30100 Murcia, Spain

**Keywords:** ellagitannin metabolism, ellagic acid, gut microbiota, urolithins, ^1^H NMR, ^13^C NMR, HSQC, urolithin G

## Abstract

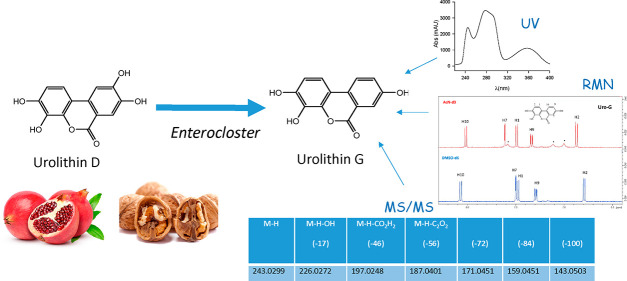

Urolithins are gut microbiota metabolites of ellagic acid. Here, we have identified and chemically characterized a novel urolithin produced from urolithin D (3,4,8,9-tetrahydroxy urolithin) by in vitro incubation with different human gut *Enterocloster* species under anaerobic conditions. Urolithin G (3,4,8-trihydroxy urolithin) was identified by ^1^H NMR, ^13^C NMR, UV, HRMS, and 2D NMR. For the identification, NMR spectra of other known urolithins were also recorded and compared. Urolithin G was present in the feces of 12% of volunteers in an overweight-obese group after consuming an ellagitannin-rich pomegranate extract. The production of urolithin G required a bacterial 9-dehydroxylase activity and was not specific to the known human urolithin metabotypes A and B. The ability to produce urolithin G could be considered an additional metabolic feature for volunteer stratification and bioactivity studies. This is the first urolithin with a catechol group in ring A while having only one hydroxyl in ring B, a unique feature not found in human and animal samples so far.

## Introduction

Ellagic acid and ellagitannins are polyphenols present in many food products, and they have been associated with biological effects that promote human health.^1^ Ellagitannins are not absorbed in the small intestine^2^ and are hydrolyzed by probiotic strains to release ellagic acid.^3^ Ellagic acid is also poorly absorbed and reaches the gut in significant amounts.^2^ In the gut, ellagic acid is converted by the gut microbiota into urolithins after lactone ring opening, decarboxylation, and sequential losses of hydroxyls to reach the final metabolites urolithin A (3,8-dihydroxy urolithin) (Uro-A), isourolithin A (3,9-dihydroxy urolithin) (IsoUro-A), and (or) urolithin B (3-hydroxy urolithin) (Uro-B).^4,5^ Other urolithin intermediates have been reported.^6^ Urolithins are much better absorbed and are bioactive metabolites with effects on metabolic syndrome, diabetes, inflammation, cardiovascular, cognitive, and muscle functions.^7−10^ The ability to produce different urolithins by *Gordonibacter* and *Ellagibacter* species is well established,^11,12^ and the whole metabolic process to reach the final circulating metabolites was recently complemented with the new strain *Enterocloster bolteae* CEBAS S4A9 and representative strains of the closest relatives (*E. bolteae* DSM 29485, DSM 15670^T^, *Enterocloster asparagiformis* DSM 15981^T^, *Enterocloster citroniae* DSM 19261^T^, *Enterocloster clostidioformis* DSM 933^T^).^13,14^

The identification of the different urolithins produced in the gut is of interest as they can be responsible for the health effects observed after the intake of food containing ellagitannins and ellagic acid. Thus, pentahydroxy, tetrahydroxy, and trihydroxy urolithins were identified as intermediate metabolites produced before reaching the final urolithins mentioned above.^4−6^ These intermediates have interest due to their potential biological effects in the gut and as intermediates in the biotechnological production of urolithins.

In the course of the elucidation of the metabolic pathways by which different bacterial strains produce urolithins, we tested the metabolism of urolithin D (3,4,8,9-tetrahydroxy urolithin) by different bacterial strains and discovered that most of the *Enterocloster* species tested yielded a potential novel urolithin which we called Urolithin G (Uro-G), which was not produced by *Gordonibacter* or *Ellagibacter* strains.^14^ We describe here the identification of this novel urolithin G.

## Material and Methods

### Chemicals

Urolithins were chemically synthesized (Villapharma, Murcia, Spain) as described elsewhere^6^ or purchased from Dalton Pharma Services (Toronto, Canada). Purity was higher than 95% in all tested compounds.

### Urolithin D Conversion by Urolithin-Producing Bacteria

The isolated strain *E. bolteae* CEBAS S4A9 and representative strains of the closest relatives (*E. bolteae* DSM 29485, DSM 15670^T^, *E. asparagiformis* DSM 15981^T^, *E. citroniae* DSM 19261^T^, *E. clostridioformis* DSM 933^T^) and other urolithin-producing bacteria (*Gordonibacter urolithinfaciens* DSM 27213^T^ and *Ellagibacter isourolithinifaciens* DSM 104140^T^) obtained from DSMZ culture collection were used to investigate their capacity to transform Uro-D as described recently.^13,14^ Briefly, 2 mL of diluted inoculum were transferred to Wilkins-Chalgren anaerobe medium (WAM, Condalab, Madrid, Spain) (20 mL), obtaining an initial load of 10^7^ CFU mL^–1^. Uro-D was dissolved in propylene glycol (PanReac Química SLU, Barcelona, Spain) and added to the 20 mL cultures to get a final concentration of 30 μM each. After incubation in an anoxic environment at 37 °C, aliquots (5 mL) were taken for HPLC analyses as described below.

### Sample Clean-Up and HPLC-DAD-MS Analyses

As previously described, aliquots (5 mL) collected during the incubation of single bacterial strains were extracted and analyzed by HPLC-DAD-ESI-Q (MS).^16^ Briefly, fermented medium (5 mL) was extracted with ethyl acetate (5 mL) (Labscan, Dublin, Ireland), acidified with 1.5% formic acid (Panreac), vortexed for 2 min, and centrifuged at 3500 *g* for 10 min. The organic phase was separated and evaporated, and the dry samples were then re-dissolved in methanol (250 μL) (Romil, Barcelona, Spain). An HPLC system (1200 Series, Agilent Technologies, Madrid, Spain) equipped with a photodiode-array detector (DAD) and a single quadrupole mass spectrometer detector in series (6120 Quadrupole, Agilent Technologies, Madrid, Spain) was used. The UV DAD was used to register UV spectra from 240 to 400 nm, and chromatograms were recorded at 305 and 360 nm.

### Isolation of the New Urolithin and Analysis of ^1^H-NMR and ^13^C-NMR Spectra

One of the chromatographic peaks, identified as an unknown urolithin, was isolated by HPLC with a semipreparative column. A higher amount of medium (200 mL) was incubated with 2 mL of a diluted inoculum of the strain CEBAS S4A9 and 30 μM of Uro-D in the conditions described above for 12 days to obtain enough amount of the isolated metabolite. The medium was extracted with the same amount (200 mL) of ethyl acetate with 1.5% formic acid. After centrifugation and evaporation, the sample was concentrated and re-dissolved in 2 mL of methanol. The sample was filtered through a 0.22 μm filter before the analysis and isolation. For this purpose, the same HPLC-DAD-ESI-SQ instrument described above was used but with a semipreparative column (Zorbax SB-C18, 9.4 × 250 mm, 5 μm). The mobile phases consisted of water +0.5% formic acid (A) and acetonitrile (B) with the following gradient: 0 min, 5% B in A; 0–4 min, 5–18% B; 4–11 min, 18–28% B; 11–19 min, 28–50% B; 19–23 min, 50–90% B; 23–24 min, 90% B; 24–25 min, 90–5% B; 25–30 min 5% B. The flow rate was 3.5 mL/min, and the injection volume was 60 μL. The new peak detected at 305 nm was manually collected to obtain the compound as pure as possible, avoiding other potential interferences. After 25 injections, the collected fractions of the new Uro-G were combined and taken to dryness in a speed vacuum concentrator. The amount of the isolated Uro-G was 56.6 mg. Then, the residue was reconstituted in 500 μL of deuterated acetonitrile (AcN-d3) for nuclear magnetic resonance (NMR) spectroscopy analysis as previously described.^15^ The AcN-d3 solution was dried under reduced pressure at 40 °C and re-dissolved in DMSO-d6 for further NMR analyses. The isolated new trihydroxy-urolithin (Uro-G) and the other urolithin standards were analyzed on a Bruker AVIII HD 500 NMR spectrometer (500.13 MHz for ^1^H and 125.77 MHz for ^13^C) equipped with a 5 mm CPP BBO cryogenic probe (Bruker Biospin, Germany).

All ^1^H-NMR spectra in ACN-d3 and DMSO-d6 were recorded at 298 K using pulse sequence noesypr1d. ^1^H spectral window was 13 ppm (6500 Hz) with chemical shift values (δ) in ppm. ^1^H NMR spectra were manually corrected for phase and baseline distortions using TOPSPIN (v3.5, Bruker Biospin). ^1^H spectra were referenced to the AcN-d3 signal (δ_H_ = 1.94 ppm) and DMSO-d6 signal (δ_H_ = 2.49 ppm).

2D NMR ^1^H-^1^H TOCSY (mlevphpr.2 pulse program) spectra were acquired for the selected sample. In TOCSY experiments, 512 transients were collected into 2048 data points for every 128 increments with a spectral width of 12 ppm (6000 Hz) for both dimensions. MLEV-17 was employed as the spin-lock scheme in the phase sensitive for the TOCSY experiments. TOCSY experiment (TPPI) was done with a mixing time of 60 ms.

All the Uro-G ^13^C-NMR spectra were also carried out in AcN-d3 and DMSO-d6. 1D-NMR ^13^C spectra were acquired using Bruker standard zgpg30 sequence (30° flip angle, bilevel 1H Waltz-16 decoupling). Acquisition time was 1.1 s and relaxation delay D1 2.00 s. ^13^C spectral window was 238.5 ppm (30,000 Hz). ^13^C spectra were referenced to CH-3 resonance of the AcN signal (δ_c_ = 1.39 ppm) and dimethyl (CH_3_)_2_ resonance of the DMSO signal (δ_c_ = 39.5 ppm).

^1^H -^13^C HSQC NMR (heteronuclear single quantum coherence) spectra were recorded using “hsqcetgpsi” pulse program (adiabatic-pulsed version) with the gradient selected sequences with 256 transients and 1024 data points for each of 512 increments. The spectral widths were 4986 Hz (from 11.2 to 1.2 ppm) for ^1^H and 23,892 Hz (from 187 to 7 ppm) for ^13^C in HSQC experiments. The data were Fourier transformed into a 4 × 2 k matrix with appropriate apodization functions. The ^1^JCH used was 145 Hz.

### UHPLC-QTOF-MS–MS Analyses

Samples were also analyzed using an Agilent 1290 Infinity UPLC system coupled to a 6550 Accurate-Mass Quadrupole time-of-flight (QTOF) (Agilent Technologies, Waldbronn, Germany). This technique provided a better identification of the new compound based on its molecular formula (obtained using mass accuracy and isotopic pattern) and the MS/MS fragmentation pattern. The chromatographic and mass spectrometric conditions tested were those previously optimized for quantifying urolithins.^16^ Briefly, separation was achieved on a reversed-phase Poroshell 120 EC-C18 column (3 × 100 mm, 2.7 μm) using the following mobile phases: water plus 0.1% formic acid (phase A) and ACN plus 0.1% formic acid (phase B) in a gradient mode. The flow rate was 0.5 mL/min, and the injection volume was 5 μL. Spectra were acquired in negative polarity with a *m/z* range of 100–1100. Besides, MS/MS parameters were optimized at a *m/z* range of 50–800 using a retention time window of 1 min, a collision energy from 20 to 40 V, and an acquisition rate of 4 spectra/s. Data were processed using the MassHunter Qualitative Analysis software (version B.10, Agilent Technologies, Waldbronn, Germany).

### Analysis of Human Fecal Samples

The samples from the POMEcardio study (NTC01916239) were obtained, as reported previously.^17^ In that study, a pomegranate extract was administered for 3 weeks to 49 overweight-obese volunteers (17 women and 32 men; BMI > 27 kg/m^2^), and feces were collected at the end. As reported, the fecal samples were extracted and analyzed by UPLC-ESI-qTOF-MS and HPLC-DAD-SQ-MS.^15,16^ The chromatograms were reanalyzed here to search for Uro-G and other new urolithins.

## Results and Discussion

### Urolithin D Conversion by Urolithin-Producing Bacteria

**Figure 1:**
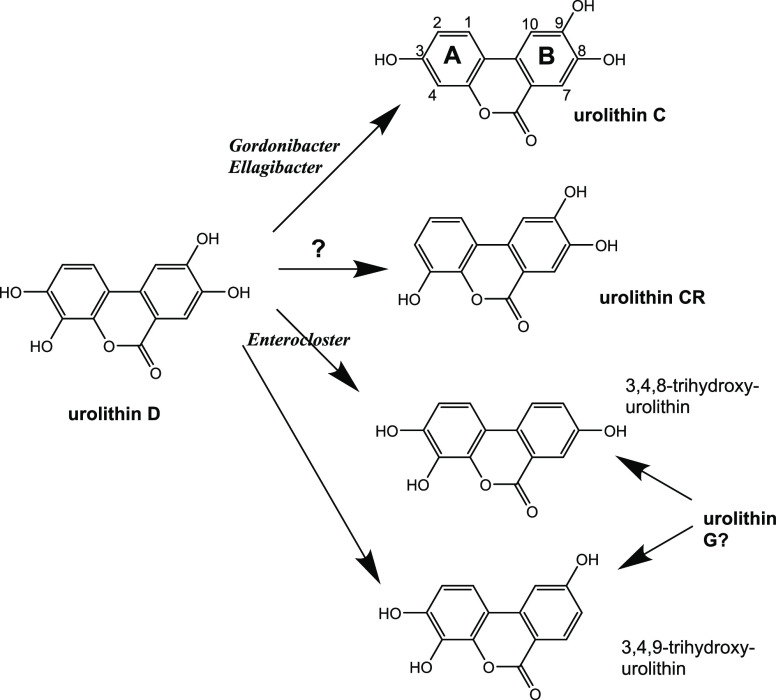
Conversion of Uro-D into other Urolithin metabolites by isolated bacterial strains under in vitro anaerobic incubation. From Uro-D, only four trihydroxy urolithins can be produced by catechol dehydroxylation reactions. Uro-C and Uro-CR have previously been reported as gut microbiota metabolites produced from ellagic acid. Only two new possible trihydroxy urolithins could be produced and only one of them is Uro-G.

**Figure 2:**
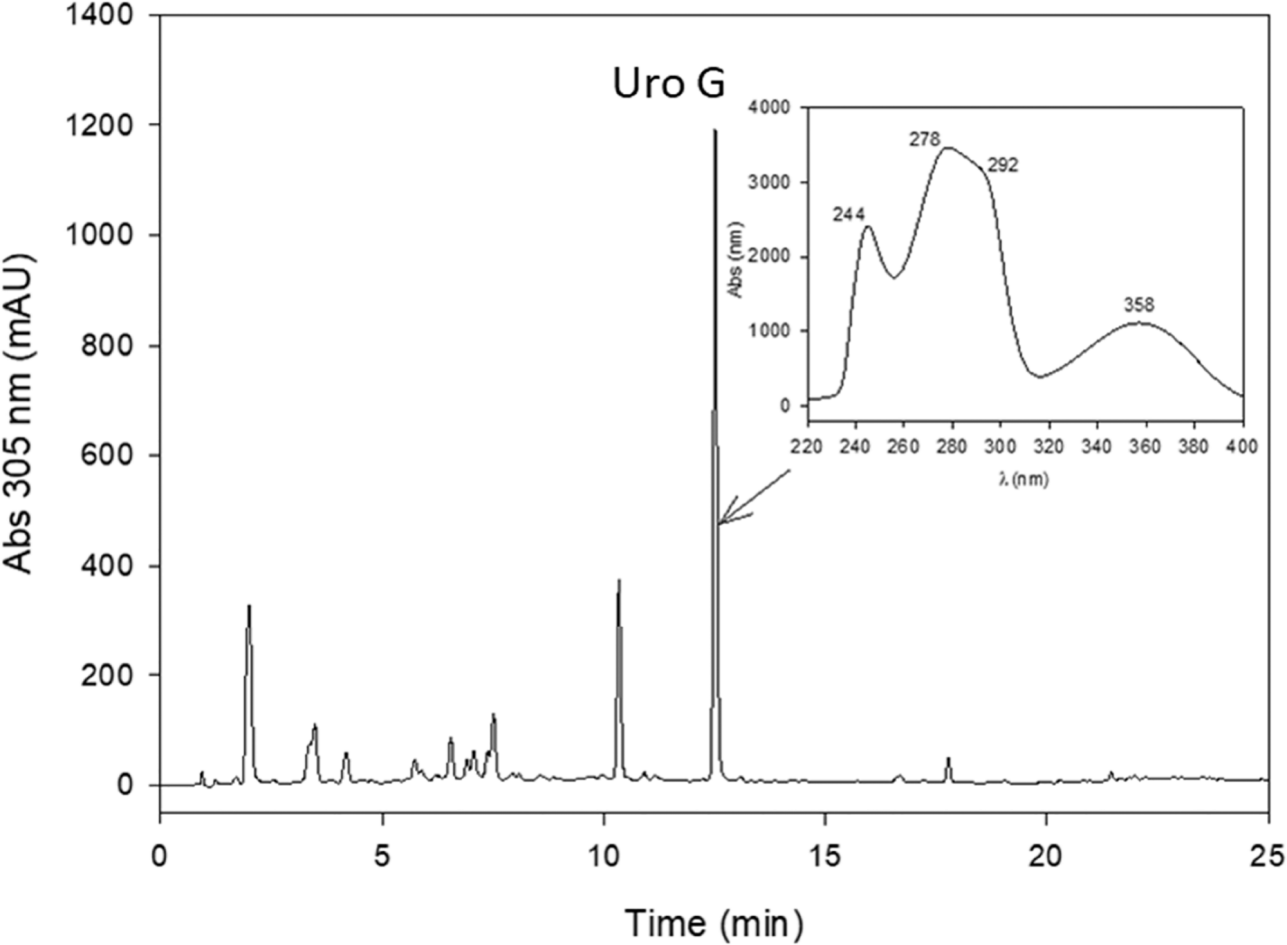
HPLC chromatogram (305 nm) of the medium, including Uro-D and *Enterocloster* 15981T, after 72 h incubation at 37 °C. Uro-D was completely converted into the novel Uro-G which has a characteristic UV spectrum.

The HPLC-DAD-SQ-MS analyses showed that Uro-D was transformed by most of the *Enterocloster* strains tested, rendering the novel trihydroxy urolithin described below with a yield of 100%. Only two of the *Enterocloster* strains tested, *E. bolteae* DSM 15679^T^ and *E. clostridioformis* DSM 933^T^, did not produce Uro-G (Table 1). In contrast, *Gordonibacter urolithinfaciens* DSM 27213^T^ and *Ellagibacter isourolithinifaciens* DSM 104140^T^ strains converted Uro-D into urolithin C (Uro-C, 3,8,9-trihydroxy urolithin), with a 54 and 100% yield, respectively (Table 1), which is a very distinctive feature. Only Uro-C, Uro-CR, 3,4,8-trihydroxy urolithin, and 3,4,9-trihydroxy urolithin could be produced by bacterial dehydroxylation of Uro-D (Figure 1). The last two trihydroxyurolithins are new metabolites not previously identified as a product of gut microbes, and only one of them that we named Urolithin G (Uro-G) was produced after incubation of Uro-D with *Enterocloster* species (Figure 2).

**Table 1:** Main Metabolites Produced and the Yield (%) after Incubating Urolithin-Producing Bacterial Strains with Uro-D (3,4,8,9-Tetrahydroxy Urolithin)^a^

strain	Uro-D
*Enterocloster bolteae* CEBAS S4A9 DSM 34392	Uro-G (100%)
*Enterocloster bolteae* DSM 15670^T^	
*Enterocloster bolteae* DSM 29485	Uro-G (100%)
*Enterocloster asparagiformis* DSM 15981^T^	Uro-G (100%)
*Enterocloster citroniae* DSM 19261^T^	Uro-G (100%)
*Enterocloster clostridioformis* DSM 933^T^	
*Gordonibacter urolithinfaciens* DSM 27213^T^	Uro-C (54%)
*Ellagibacter isourolithinifaciens* DSM 104140^T^	Uro-C (100%)

a Uro-G (3,4,8-trihydroxy urolithin); Uro-C (3,8,9-trihydroxy urolithin).

### Identification of the Novel Trihydroxy Urolithin

The unknown trihydroxy urolithin ([M – H]^−^ at *m/z* 243) showed an *R_t_* at 12.58 min that did not coincide, under the same assay conditions, with the already known trihydroxy urolithins, i.e., Uro-M7 (3,8,10-trihydroxy urolithin) (*R_t_* 13.59 min), Uro-C (*R_t_* 12.44 min), Uro-M7R (4,8,10-trihydroxy urolithin) (*R_t_* 14.19 min), and Uro-CR (4,8,9-trihydroxy urolithin) (*R_t_* 13.17 min),^15^ suggesting a new metabolite (urolithin G) (Figure 2). UPLC-QTOF-MS analyses showed a molecular formula of C_13_H_8_O_5_ with a score of 98.57 and an error of −1.47 for that metabolite, coincident with a trihydroxy urolithin. Its MS–MS fragmentation was similar to those of other trihydroxy urolithins with no characteristic fragments with a diagnostic value for metabolite discrimination (Table 2). To identify the new Uro-G, the ^1^H NMR spectrum was recorded in AcN-d3 as this solvent is easily removed by reduced pressure concentration at low temperature (40 °C), which allows the fast recovery of the isolated metabolite for further analyses and use as a standard and for biological assays. The spectrum in this solvent also showed the protons of the three phenolic hydroxyls (Figure S1). However, the spectrum was not discriminant between the two possibilities (Figure 1) since (i) the estimation obtained in the ChemDraw software was based on spectra recorded in DMSO-d6 (Table 3) and (ii) the solubility of the isolated urolithin in acetonitrile was not sufficient for some urolithins. Therefore, we also recorded the ^1^H NMR spectra in DMSO-d6 (Table 3). The NMR spectra in both solvents were consistent with the spectra of a trihydroxy urolithin showing five aromatic H signals,^15^ with those of other available urolithin standards (Supporting Figure S1), and with the data reported in previous publications on NMR analysis of urolithins.^18−22^

**Figure 3:**
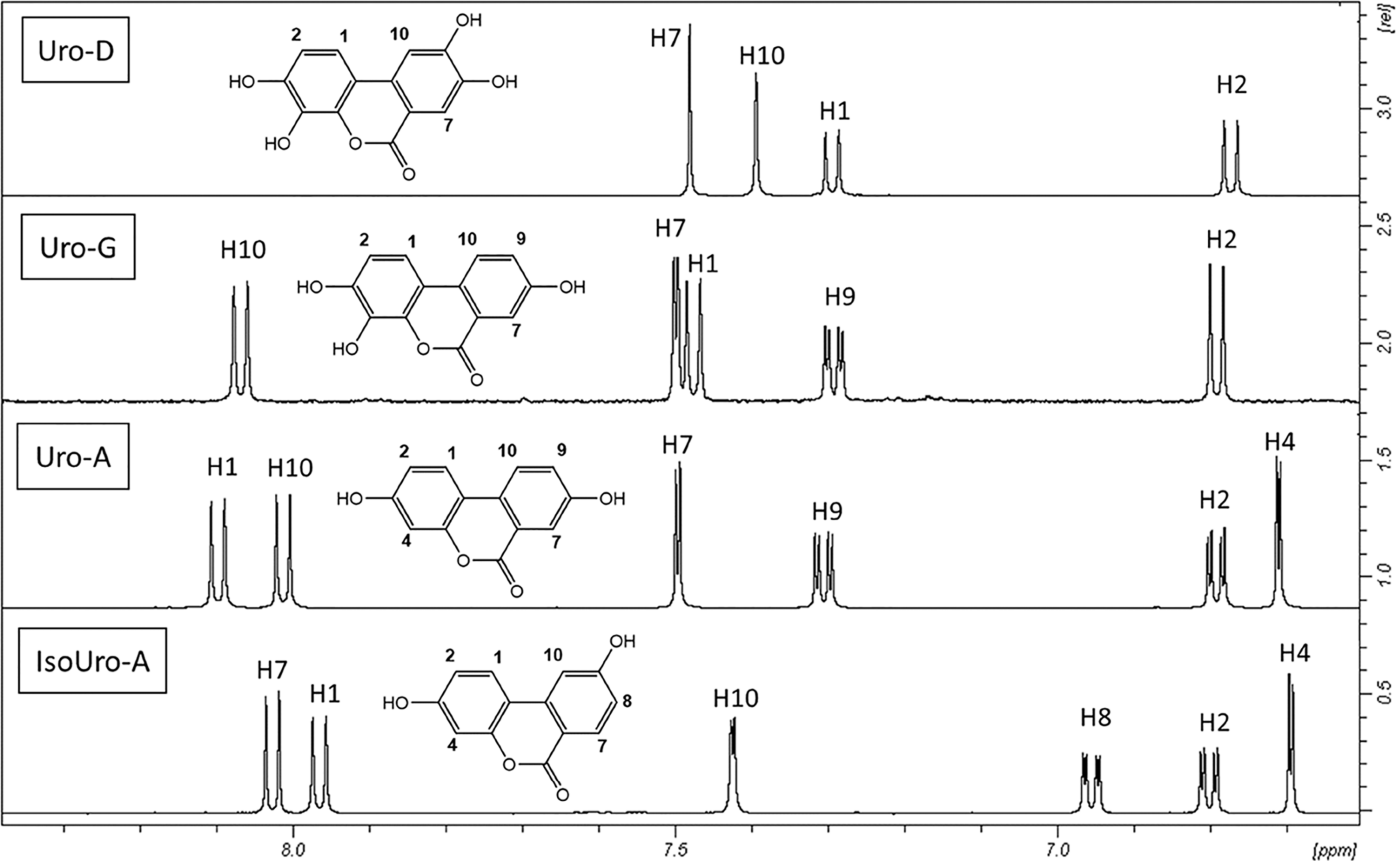
^1^H NMR spectra in DMSO-d6 of Uro-G, Uro-D, Uro-A, and IsiUro-A. Five clear proton signals are observed. The spectra show that Uro-G has protons in ring A similar to those of Uro-D and protons in ring B similar to those of Uro-A.

**Table 2:** UHPLC-QTOF-MS/MS Fragments of the New Urolithin G and Other Trihydroxy Urolithins at 30 V Collision Energy^a^

urolithins	M–H	M–H–OH (−17)	M–H–CO (−28)	M–H–CO_2_ (−44)	M–H–CO_2_H (−45)	M–H–CO_2_H_2_ (−46)	M–H–C_2_O_2_ (−56)	M–H–C_3_O_2_H_2_ (−70)	(−72)	(−84)	(−96)	(−100)
	C_13_H_8_O_5_		C_12_H_7_O_4_	C_12_H_7_O_3_		C_12_H_5_O_3_	C_11_H_7_O_3_	C_10_H_5_O_3_	C_11_H_7_O_2_	C_10_H_7_O_2_	C_9_H_7_O_2_	C_10_H_7_O
Uro-C	243.0299	226.0275	215.0355	199.0399			**187.0398**		171.0438			143.0495
Uro-M7	243.0299				198.0332		187.0400	173.0239			**147.0452**	
Uro-CR	243.0299	**226.0276**	215.0353			197.0251	**187.0402**		171.0438			
Uro-M7R	243.0299	226.0278	215.0336	**199.0359**			187.0401		171.0454			**143.052**
Uro-G	243.0299	226.0272				**197.0248**	187.0401		171.0451	159.0451		143.0503

a Bold represents the most intense fragments.

**Table 3:** ^1^H NMR Analyses of Urolithins Dissolved in AcN-d3 and DMSO-d6 Compared with the Estimated Values in ChemDraw Using DMSO-d6

metabolite	H-1	H-2	H-4	H-7	H-8	H-9	H-10
**AcN-d3**							
Uro-D 3,4,8,9-OH	d7.41, *J* = 8.8 Hz	d6.85, *J* = 8.8 Hz		s7.60			s7.48
Uro-C 3,8,9-OH	d7.865 *J* = 9,1 Hz	dd6.83 *J* = 2.7, 9.1 Hz	d6.77 *J* = 2.7 Hz	s7.60			s7.48
Uro-M7 3,8,10-OH	d8.75 *J* = 9 Hz	dd6.81 *J* = 2,4, 9 H	d6.76 *J* = 2.4 Hz	d7.27 *J* = 2.4		d6.84 *J* = 2.4	
Uro-A 3,8-OH	d8.1 *J* = 8.8 Hz	dd6.82 *J* = 2.7, 8.7 Hz	d6.79 *J* = 2.7 Hz	d7.60, *J* = 2.7 Hz		dd7.345 *J* = 2,7 Hz; 8.7 Hz	d7.945 *J* = 8.8 Hz
IsoUro-A 3,9-OH	d7.94, *J* = 8.6 Hz	dd6.88 *J* = 2.3, 8.6 Hz	d6.79 *J* = 2.3 Hz	8.13 *J* = 8.6 Hz	dd7.02, *J* = 2.3, 8.6 Hz		d7.47 *J* = 2.3 Hz
Uro-G 3,4,8-OH	d7.49 *J* = 8.75	d6.87 *J* = 8.70		d7.61 *J* = 2.7		dd7.34 *J* = 2,76, 8.75	d8.01 *J* = 8.8 Hz
**DMSO-d6**							
Uro-D 3,4,8,9-OH	d7.29, *J* = 8.8 Hz	d6.77, *J* = 8.8 Hz		s7.48			s7.39
Uro-C 3,8,9-OH	d7.83 *J* = 9,1 Hz	dd6.77 *J* = 2.7,9.1 Hz	d6.67 *J* = 2.7 Hz	s7.47			s7.42
Uro-M7 3,8,10-OH	d8.73 *J* = 9 Hz	dd6.74 *J* = 2.4, 9 H	d6.68 *J* = 2.4 Hz	d7.11 *J* = 2.4		d6.84 *J* = 2.4	
Uro-A 3,8-OH	d8.10 *J* = 8.8 Hz	dd6.79 *J* = 2.7, 8.7 Hz	d6.71 *J* = 2.7 Hz	d7.49 *J* = 2.7 Hz		dd7.31 *J* = 2.7 Hz; 8.7 Hz	d8.01 *J* = 8.8 Hz
IsoUro-A 3,9-OH	d7.96 *J* = 8.6 Hz	dd6.79 *J* = 2.3, 8.6 Hz	d6.69 *J* = 2.3 Hz	d8.02 *J* = 8.6 Hz	dd6.95, *J* = 2.3, 8.6 Hz		d7.42 *J* = 2.3 Hz
Uro-G 3,4,8-OH	d7.48 *J* = 8.75	d6.79 *J* = 8.70		d7.50 *J* = 2.7		dd7.31 *J* = 2.76, 8.75	D8.08 *J* = 8.8 Hz
**ChemDraw estimated (DMSO-d6)**							
Uro-D 3,4,8,9-OH	d7.23, *J* = 8.8 Hz	d6.73, *J* = 8.8 Hz		s7.55			s7.12
Uro-C 3,8,9-OH	d7.67 *J* = 9.1 Hz	dd6.76 *J* = 2.7,9.1 Hz	d6.88 *J* = 2.7 Hz	s7.55			s7.12
Uro-M7 3,8,10-OH	D7.67 *J* = 9 Hz	dd6.76 *J* = 2.4, 9 H	d6.85 *J* = 2.4 Hz	d7.28 *J* = 2.4		d6.58 *J* = 2.4	
Uro-A 3,8-OH	D7.67 *J* = 8.8 Hz	dd6.76 *J* = 2.7, 8.7 Hz	d6.88 *J* = 2.7 Hz	d7.72 *J* = 2.7 Hz		dd7.11 *J* = 2.7 Hz; 8.7 Hz	d7.75 *J* = 8.8 Hz
IsoUro-A 3,9-OH	d7.67 *J* = 8.6 Hz	dd6.76 *J* = 2.3, 8.6 Hz	d6.88 *J* = 2.3 Hz	d8.20 *J* = 8.6 Hz	dd6.95, *J* = 2.3, 8.6 Hz		d7.29 *J* = 2.3 Hz
Uro-G 3,4,8-OH	d7.23 *J* = 8.75	d6.73 *J* = 8.70		d7.72 *J* = 2.7		dd7.11 *J* = 2.76, 8.75	D7.75 *J* = 8.8 Hz
IsoUro-G 3,4,9-OH	d7.23 *J* = 8.75	d6.73 *J* = 8.70		D8.20 *J* = 2.7	dd6.95 *J* = 2.76, 8.75		D7.29 *J* = 2.7 Hz

The ^1^H NMR spectra in DMSO-d6 of Uro-D, Uro-A, and IsoUro-A were compared with the spectrum of Uro-G (Figure 3). Uro-G showed very similar chemical shifts for H1 and H2 than Uro-D suggesting a similar hydroxylation pattern in ring A with hydroxyls at 3 and 4 positions. In addition, Uro-G showed almost identical chemical shifts for the H7, H9, and H10 as Uro-A and very different from those of IsoUro-A, supporting a Uro-A hydroxylation pattern for the ring B.

**Figure 4:**
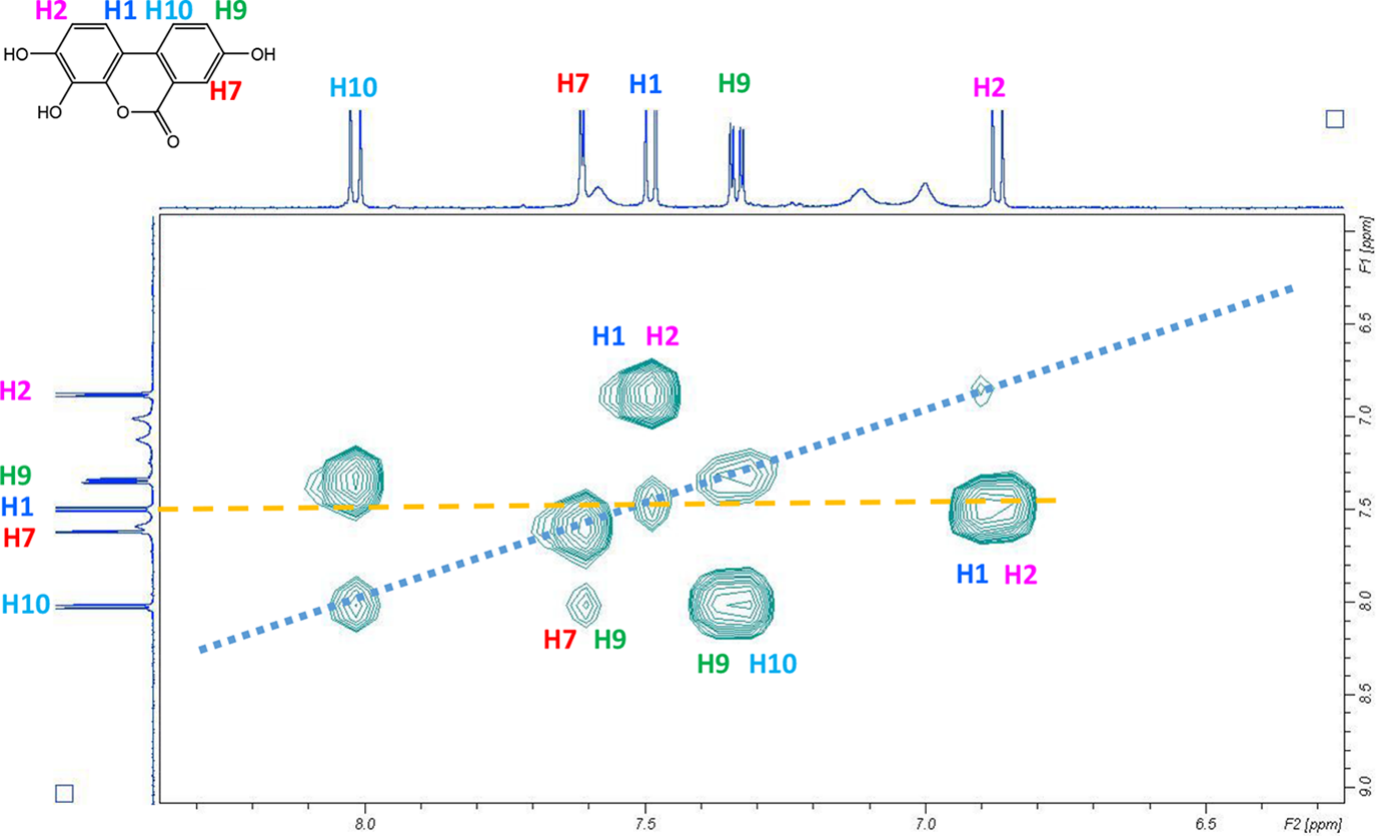
^1^H NMR TOCSY H-H 2D spectrum of Uro-G dissolved in AcN-d3. Short distance (H1–H2 and H9–H10) and long distance (H7–H9) couplings are shown.

The TOCSY experiment (Figure 4) clearly showed the coupling of H1 and H2, and H9 with H10, and the long-distance coupling of H-9 and H7. Uro-G does not have a singlet for H-7, confirming that this is not Uro-C or Uro-CR. In addition, Uro-G does not have a ^1^H NMR signal at 7.02 ppm for H-8, which should be characteristic of the spectrum of the 3,4,9-trihydroxy urolithin isomer (ChemDraw estimation in DMSO-d6, Table 3).

**Figure 5:**
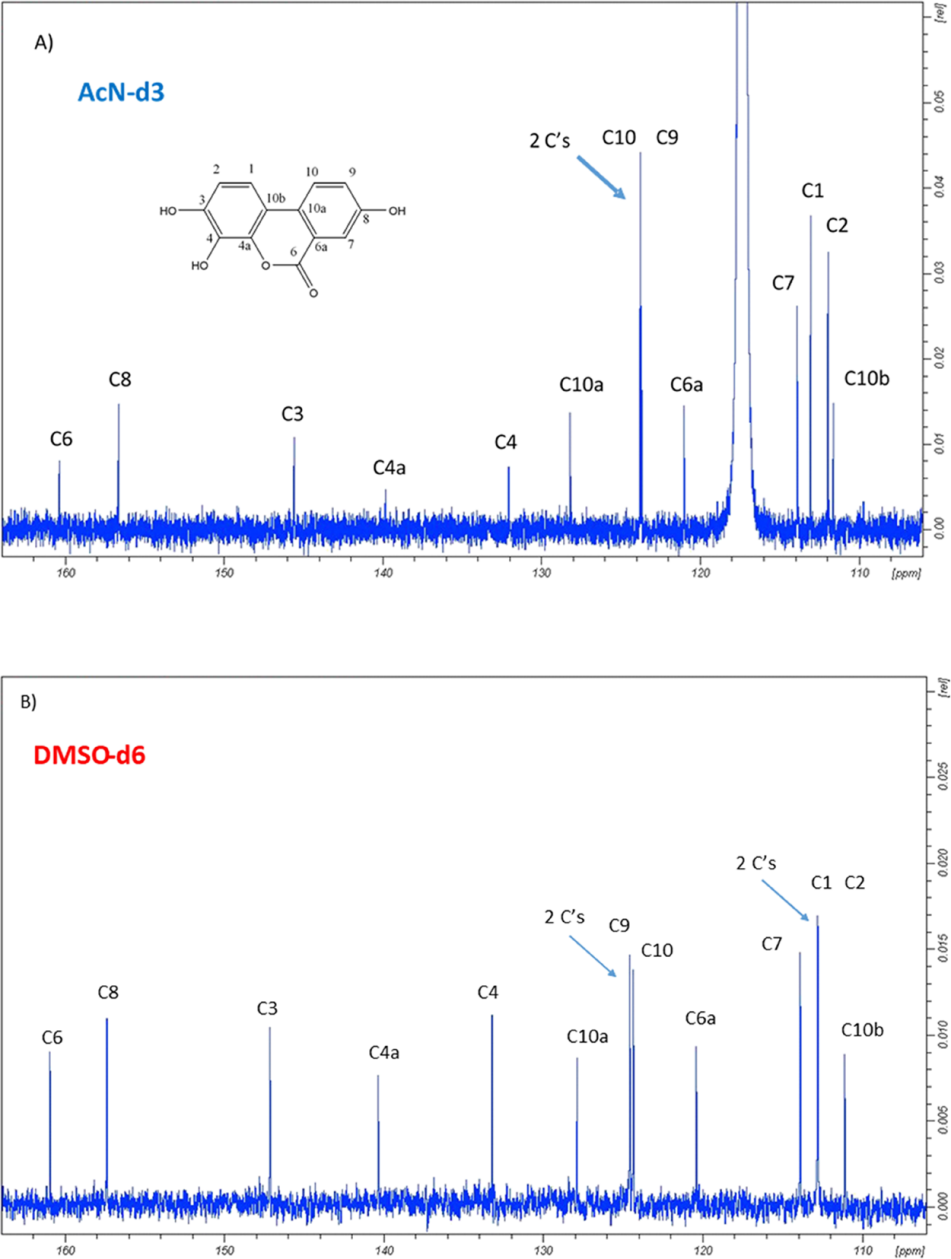
^13^C NMR spectra of Uro-G dissolved in AcN-d3 (A) and DMSO-d6 (B). Carbon assignments in both deuterated solvents.

The ^13^C NMR was also consistent with the proposed structure of 3,4,8-trihydroxy urolithin for Uro-G, both in AcN-d3 and DMSO-d6 (Figure 5).

**Figure 6:**
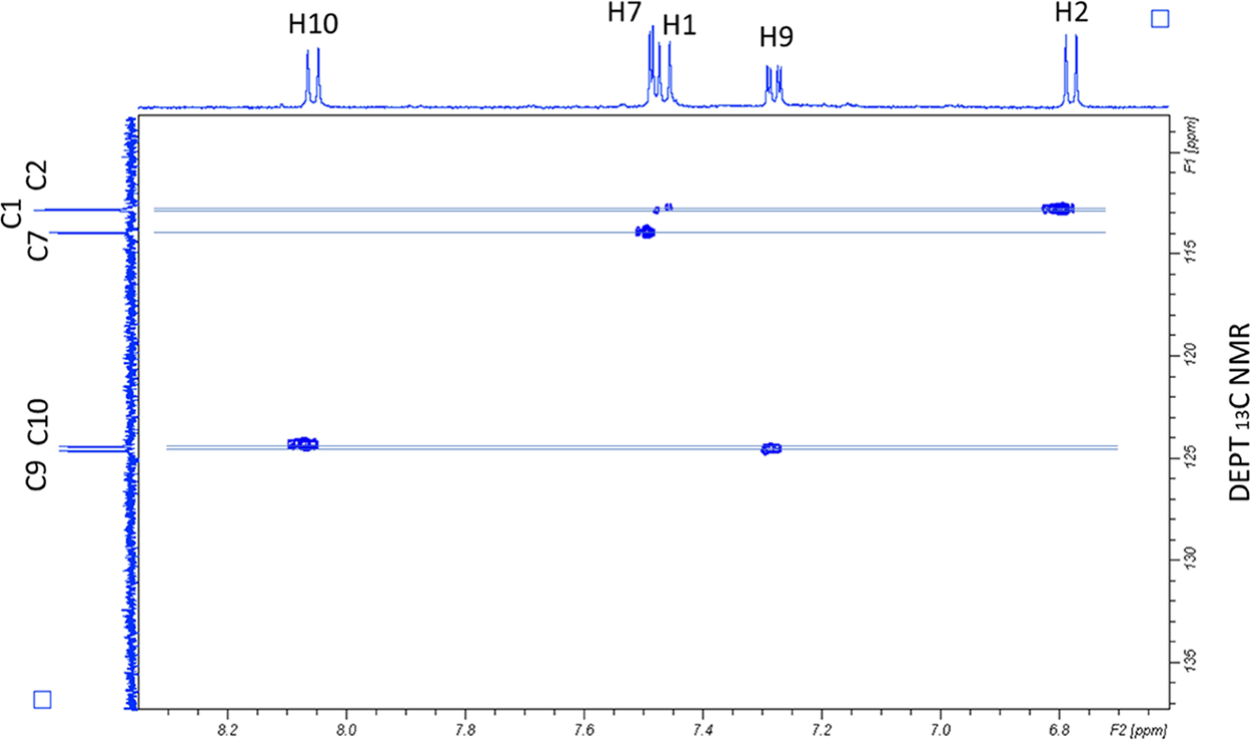
HSQC analysis of Uro-G. Connections of the five C–H carbons in the DEPT ^13^C NMR and the five protons in the ^1^H NMR spectra.

The HSQC experiment also confirmed the Uro-G structure. In Figure 6, we show the HSQC results in which the five C–H carbons (DEPT) and the connected five protons of Uro-G are evidenced. The results also confirmed the ^13^C NMR assignments (Figure 5).

**Figure 7:**
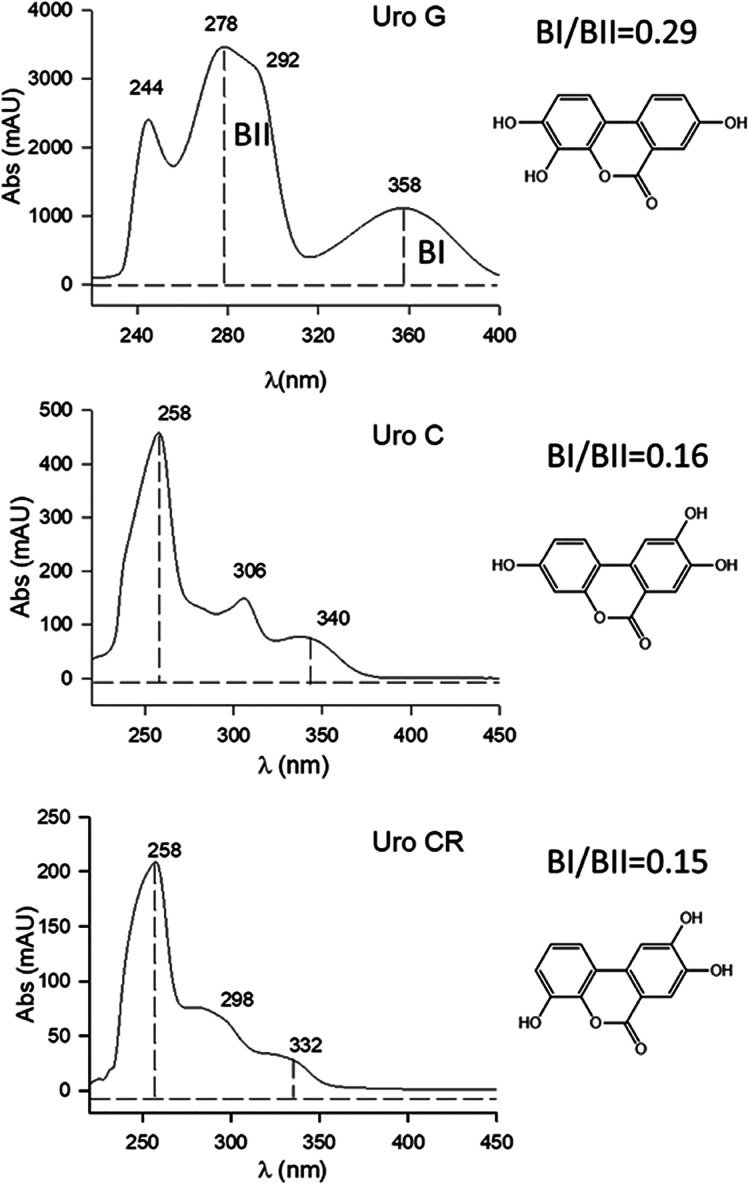
UV spectra of Uro-G and the other tri-hydroxy urolithins that could be produced from Uro-D by dehydroxylation and of the authentic standards available. BI/BII ratios were calculated as a diagnostic feature for the presence/absence of hydroxyl at the 9-position.^15^

The UV spectrum of Uro-G (Figure 7) with a BI/BII ratio of 0.29 also indicated the lack of hydroxyl at the 9-position of the urolithin nucleus, in contrast with the 9-hydroxy urolithins Uro-C (BI/BII 0.16) and Uro-CR (BI/BII 0.15) and in agreement with previous studies.^15^ The other feasible isomer, 3,4,9-trihydroxy urolithin, should have a UV spectrum with a BI/BII ratio around 0.15, and thus, the UV confirmed the structure of Uro-G as 3,4,8-trihydroxy urolithin.

Uro-G was only obtained after Uro-D incubation with *Enterocloster* species that harbor 9-dehydroxylase activity.^14^

In this study, we have only considered dehydroxylations of Uro-D by the dehydroxylase enzymes present in the bacteria used (*Gordonibacter*, *Ellagibacter*, and *Enterocloster*) (Figure 1). Other metabolites could have been considered to be produced from Uro-D by hydroxyl transfer as it has been reported for the conversion of pyrogallol (1,2,3-trihydroxy benzene) into phloroglucinol (1,3,5-trihydroxy benzene) by the anaerobic bacteria *Pelobacter acidogallici*(23) and *Eubacterium oxidoreducens*,^24^ although this is very unlikely and has not been reported for the bacteria assayed in the present study.

**Figure 8:**
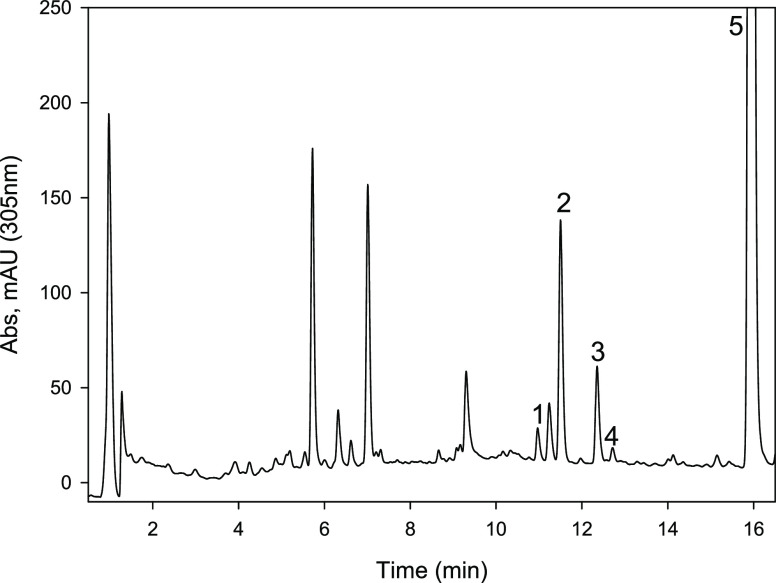
HPLC chromatograms (305 nm) of fecal samples from a volunteer after consuming an ellagitannin-rich pomegranate extract. (1) ellagic acid; (2) Uro-M6 (3,8,9,10-tetrahydroxy urolithin); (3) Uro-C (3,8,9-trihydroxy urolithin); (4) Uro-G (3,4,8-trihydroxy urolithin); (5) Uro-A (3,8-dihydroxy urolithin).

For the first time, we have described the spectroscopic features of the new Uro-G produced from Uro-D in vitro. Therefore, the occurrence of this metabolite in human feces after the intake of ellagitannins would confirm the activity of human *Enterocloster* species in vivo. For this purpose, we revisited the analyses of human feces after the intake of pomegranate ellagitannins in the POMEcardio study.^17^ This survey confirmed the occurrence of Uro-G in some of the fecal samples (6 out of 49 volunteers; 12%) (Figure 8). However, Uro-G was a minor metabolite in the feces of volunteers belonging to both metabotypes A and B, and thus, its occurrence was not specifically associated with one of these urolithin-producing metabotypes.

This new urolithin could also be present in human biological fluids (plasma and urine) since some trihydroxy urolithin derivatives, such as Uro-C, have been detected in some cases.^25^ However, this was not addressed in the present study.

To the best of our knowledge, Uro-G is the first urolithin with a catechol group in the A ring while having only one hydroxyl in the B ring, a unique feature not found in human and animal samples so far.

## Supporting Information Available

Figure S1. ^1^H NMR spectra of urolithin D, urolithin C, urolithin M7, urolithin G, urolithin A, and isourolithin A in AcN-d3 and in DMSO-d6. *Signals from hydroxyl protons; Figure S2. ^13^C NMR spectrum (zoom from *d* = 110–115 ppm) of urolithin G in AcN-d3 and DMSO-d6. C2 and C7 were clearly separated in AcN-d3, while they appeared very close in the DMSO-d6 spectrum (PDF)
