# Supplementary material for: NMR Spectroscopic Identification of Urolithin G, a Novel Trihydroxy Urolithin Produced by Human Intestinal Enterocloster Species
Source: J Agric Food Chem. 2023 Jul 26;71(31):11921–8. doi: 10.1021/acs.jafc.3c01675 (PMC10416303; doi:10.1021/acs.jafc.3c01675)

Supporting Information

NMR Spectroscopic Identification of Urolithin G, a Novel Trihydroxy Urolithin Produced by Human Intestinal *Enterocloster* Species.

*David Beltrán,[1] María D. Frutos-Lisón[1], Rocío García-Villalba, [1] José E. Yuste, [2] Victor García, [2] Juan C. Espín, [1] María V. Selma, [1] Francisco A. Tomás-Barberán[1]**

[1] Quality, Safety and Bioactivity of Plant-Derived Foods, CEBAS-CSIC, University Campus, Edif. 25, Espinardo, 30100 Murcia, Spain.

[2] Metabolomics Unit. CEBAS-CSIC, 30100 Murcia, Spain

*Email: fatomas@cebas.csic.es

Figure S1

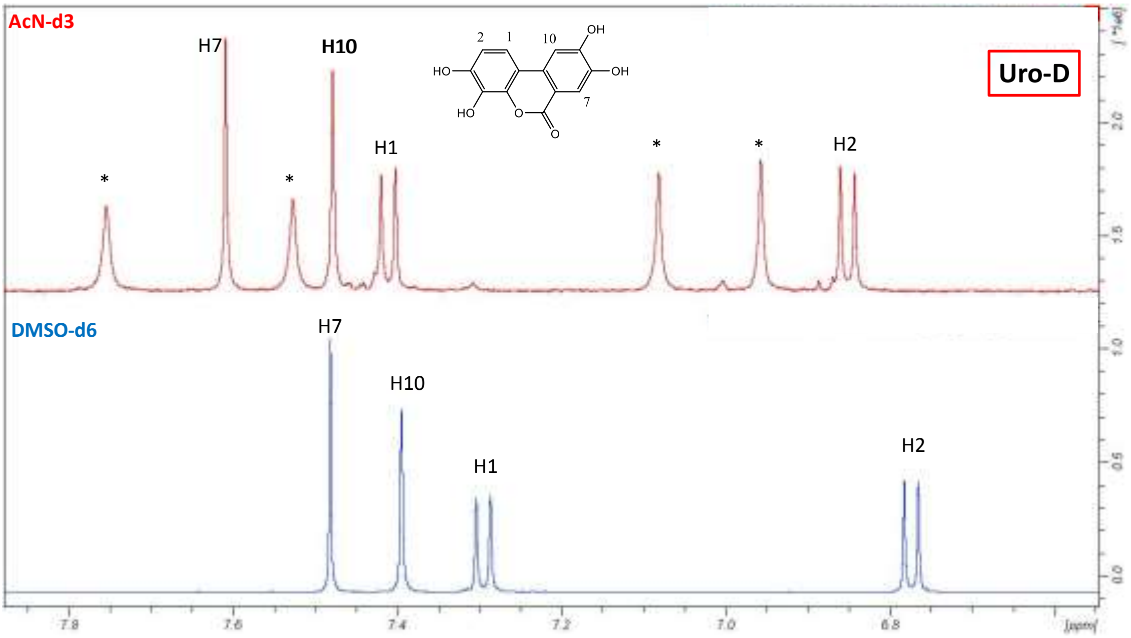
AcN-d3
H7
H10
H1
H2
*
DMSO-d6
H7
H10
H1
H2
HO
HO
OH
OH
O
O
2
1
10
7
Uro-D
7.8
7.6
7.4
7.2
7.0
6.8
ppm

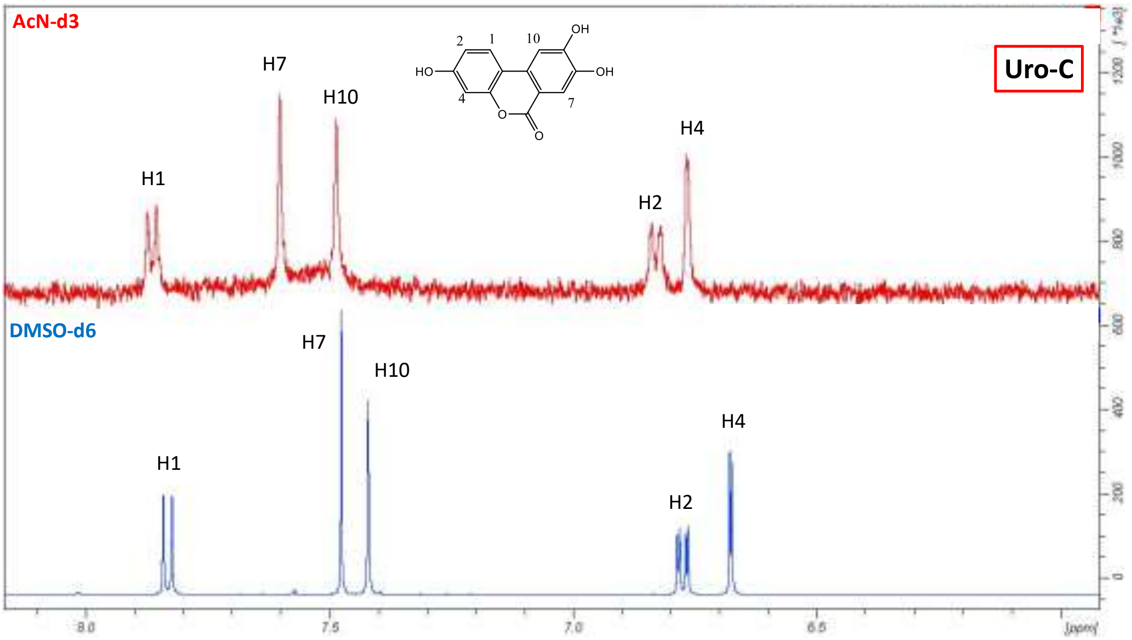
AcN-d3
H1
H7
H10
H2
H4
DMSO-d6
H7
H10
H1
H2
H4
HO
OH
OH
O
O
2
1
10
4
7
Uro-C
8.0
7.5
7.0
6.5
ppm

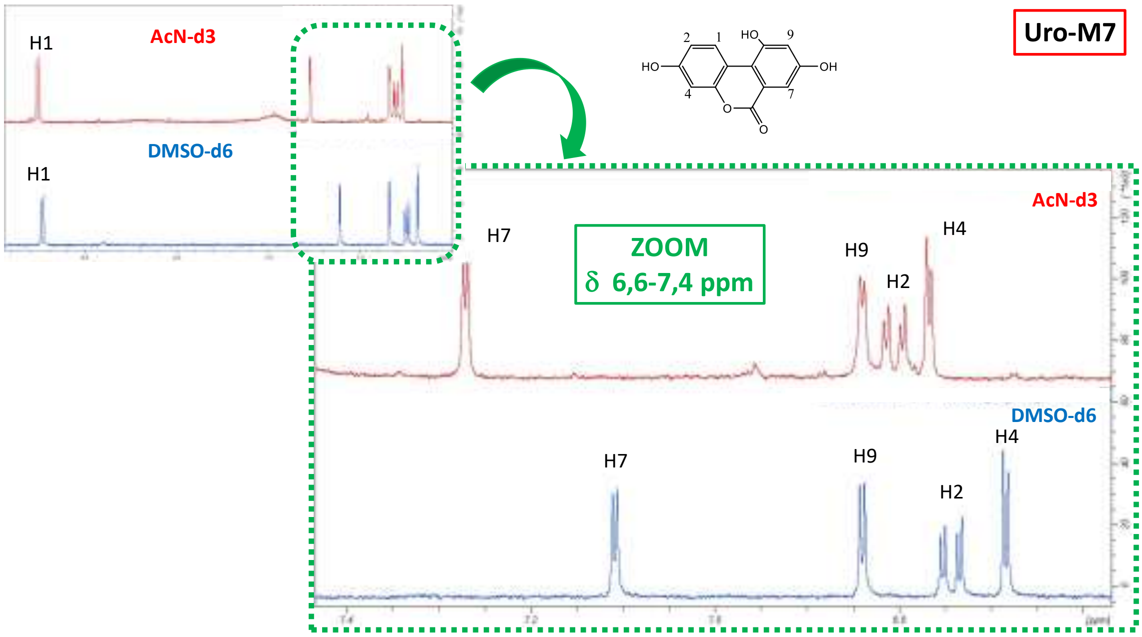
Uro-M7
AcN-d3
DMSO-d6
H1
H1
HO
OH
HO
ZOOM
δ 6,6-7,4 ppm
AcN-d3
H7
H9
H2
H4
DMSO-d6
H7
H9
H2
H4

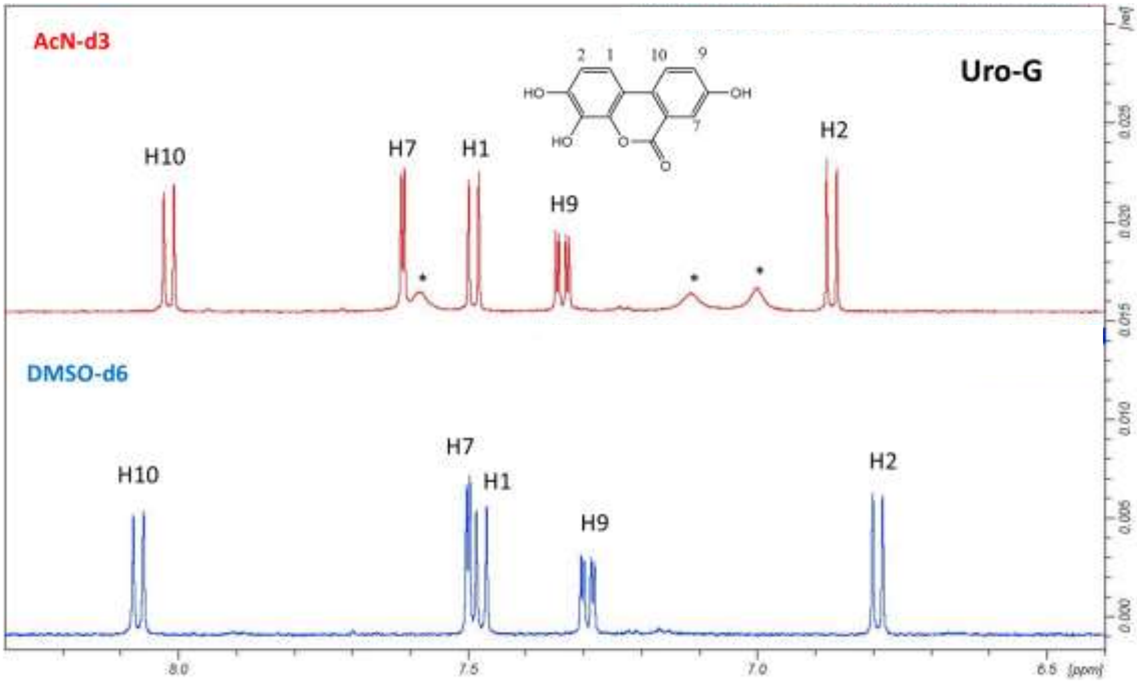
AcN-d3
Uro-G
HO
OH
HO
H10
H7
H1
H9
H2
DMSO-d6
H10
H7
H1
H9
H2
8.0
7.5
7.0
6.5

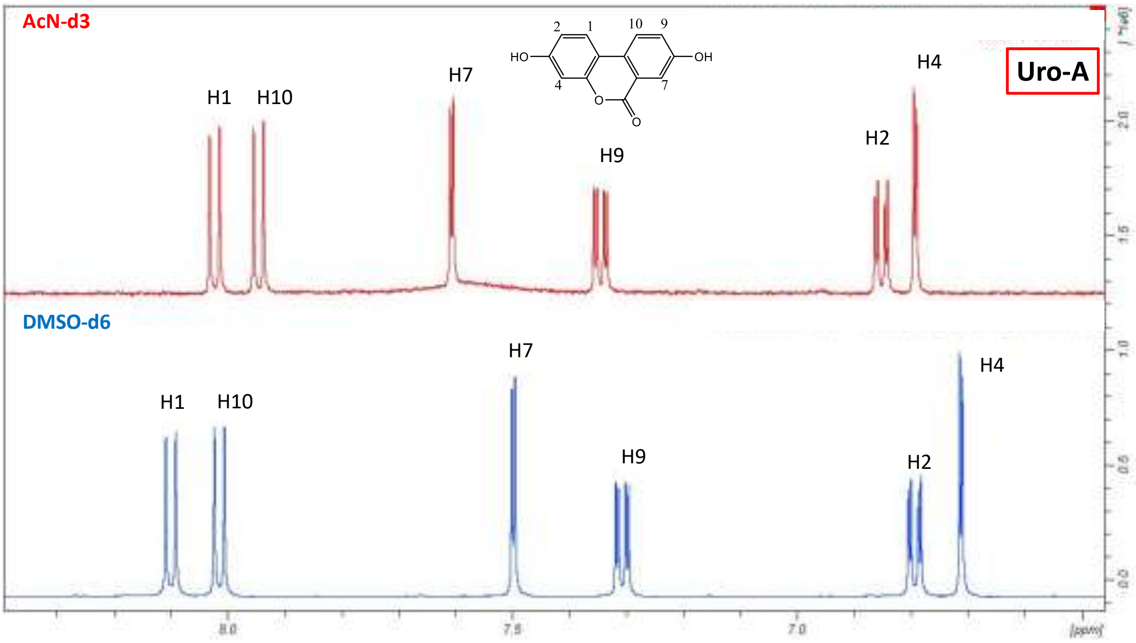
AcN-d3
Uro-A
H1
H10
H7
H9
H2
H4
DMSO-d6
H1
H10
H7
H9
H2
H4
8.0
7.5
7.0
[ppm]

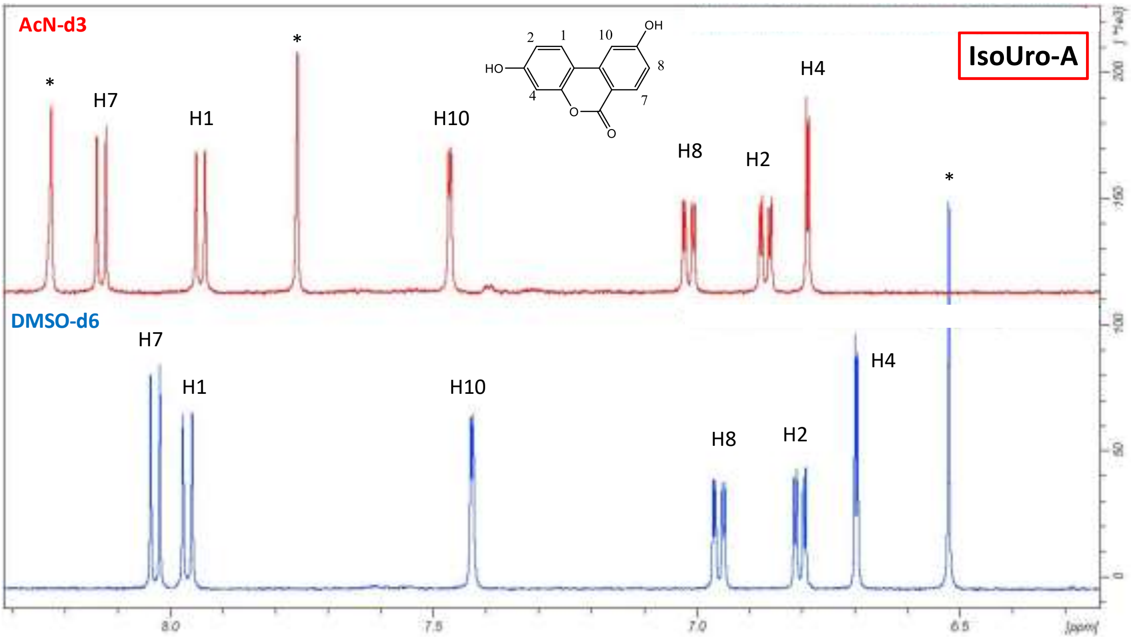
AcN-d3
IsoUro-A
*
H7
H1
*
H10
H8
H2
H4
*
DMSO-d6
H7
H1
H10
H8
H2
H4
8.0
7.5
7.0
6.5
[ppm]

Figure S2

Comparative ^{13}C-NMR of Uro-G: δ = 110-115 ppm

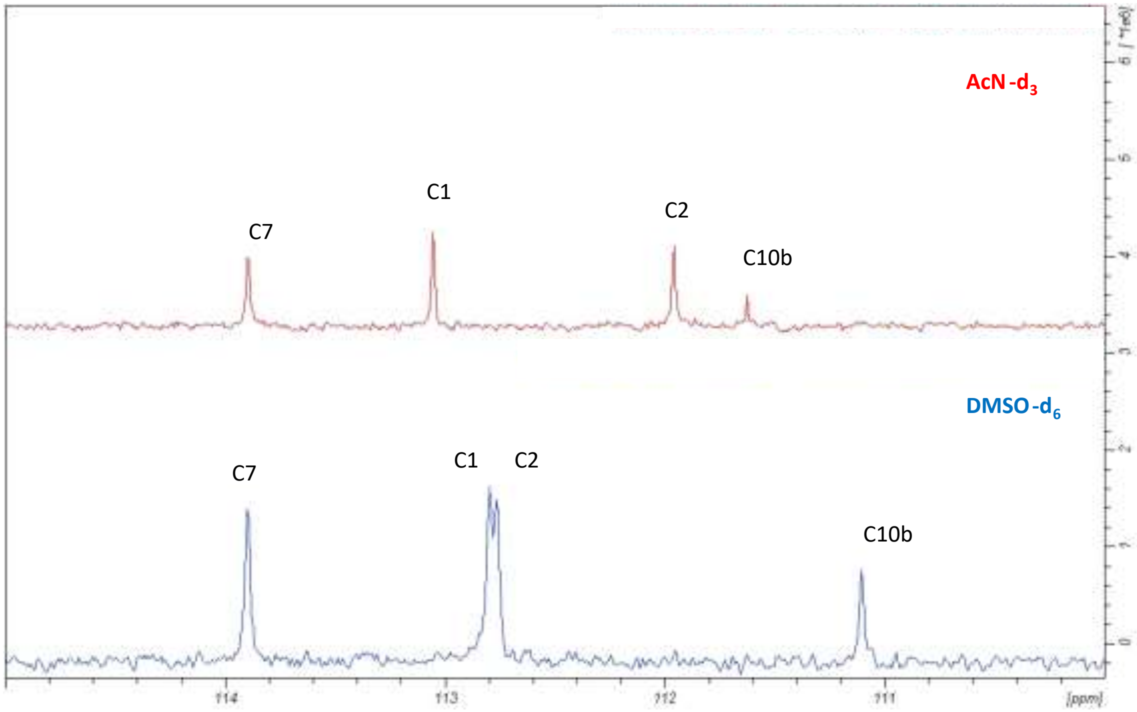

Supplement: Supplementary file 1 — jf3c01675_si_001.pdf [file jf3c01675_si_001.pdf]
